# Gender-related differences in migraine

**DOI:** 10.1007/s10072-020-04643-8

**Published:** 2020-08-26

**Authors:** Gianni Allais, Giulia Chiarle, Silvia Sinigaglia, Gisella Airola, Paola Schiapparelli, Chiara Benedetto

**Affiliations:** Department of Surgical Sciences, Women’s Headache Center, University of Turin, Via Ventimiglia 3, 10126 Turin, Italy

**Keywords:** Gender, Migraine, Neuromodulation, Progesterone, 17 beta-estradiol

## Abstract

Migraine is considered mostly a woman’s complaint, even if it affects also men. Epidemiological data show a higher incidence of the disease in women, starting from puberty throughout life. The sex-related differences of migraine hold clinical relevance too. The frequency, duration, and disability of attacks tend to be higher in women. Because of this, probably, they also consult specialists more frequently and take more prescription drugs than men. Different mechanisms have been evaluated to explain these differences. Hormonal milieu and its modulation of neuronal and vascular reactivity is probably one of the most important aspects. Estrogens and progesterone regulate a host of biological functions through two mechanisms: nongenomic and genomic. They influence several neuromediators and neurotransmitters, and they may cause functional and structural differences in several brain regions, involved in migraine pathogenesis. In addition to their central action, sex hormones exert rapid modulation of vascular tone. The resulting specific sex phenotype should be considered during clinical management and experimental studies.

## Introduction

Migraine is generally considered a woman’s complaint owing to its preponderance among women and the greater healthcare resource use by women for migraine headache. Epidemiological data subdivide the prevalence of migraine by age group. In prepubertal children, the 1-year prevalence is roughly the same for boys and girls (overall range, 2–5%; 2.4% and 2.5% in girls and boys, respectively, aged 7–9 years; 5.4% and 3.9% in girls and boys, respectively, aged 10–12 years) [1]. Starting at puberty with its accompanying hormonal changes, the prevalence increases in both sexes, and it is higher in girls than boys from 13 to 15 years (6.4% and 4.0%, respectively). This sex-related difference in prevalence remains throughout life. Between 10 and 20% of women report experiencing their first migraine attack at the start of menarche [2]. Migraine incidence peaks first at around age 35 years (25–30% of women and 8% of men) [3] and then again at around age 50, after which it declines with advancing age [4]. The elderly suffer less frequently from migraine but more often from secondary headache than young people. The decline in migraine frequency is proportional to advancing age, starting at the sixth/seventh decade, and further decreases after age 75. Nevertheless, new onset migraine after age 65 is noted to occur in 0.5% of the population [5].

The American Migraine Prevalence and Prevention (AMPP) study reported a cumulative incidence of lifetime migraine in 43% of women and 18% of men [6], a slightly lower rate than more recent data reveal, according to which the risk of migraine is 3.25 times higher for women than for men [4, 7]. The estimated prevalence of migraine in the Italian population is 32.9% in women and 13.0% in men [4]. A recent national survey of pharmacies in Italy revealed a prevalence of 43% of “definite migraine” (defined by the ID Migraine questionnaire) [7] in a female-to-male ratio of 4.9: 1 [8]. These survey findings show that migraine is much more common in the general population than the data from public healthcare databases would indicate; the reason is that many migraine sufferers choose to self-medicate rather than consult a headache specialist for diagnosis and treatment.

## Clinical characteristics of migraine in men and women

The sex-related differences of migraine hold epidemiological and clinical relevance. Most studies agree that there are no sex-related differences in attack frequency: 1 to 4 monthly headache days (MHD) on average for both sexes (48.8% in women and 45.3% in men) [4, 9, 10]. Bolay reported that high frequency migraine (> 10 MHD) is more common among men than women (16.7% and 14.9%, respectively; prevalence rate [PR] 0.90; 95% confidence interval [CI] 0.83–0.97). Attack frequency differs between age groups: migraine without aura (MO) is higher among 18–29-year-olds than among 40–49-year-olds (*p* < 0.0001) and after age 50 (*p* = 0.0013). Similarly, migraine with aura (MA) is more frequent in women aged 18 to 29 years than in those aged 30 to 39 years (*p* = 0.0308). This difference in attack frequency between age groups is significant for women but not for men migraineurs [10]. Attack duration of MO and MA is also age-related: shorter in 18–29-year-olds and longer in women aged 30 and over (*p* = 0.0407 for MO and *p* = 0.0043 for MA).

Most studies report longer attack duration in women than in men (28.4 h in men and 36.7 h in women; *p* = 0.01) and a higher recurrence rate. Study findings diverge on whether attack intensity differs between the sexes [4, 10–13]. Because the perception of pain intensity is subjective, whereas attack duration is an objective measure, some researchers report that, at equal pain scores for men and women, the longer duration of attacks and recovery time may be the reasons why women grade their pain intensity higher than men. Added to this is the commonplace belief that, because of gender roles, men are less likely to rate their perception of pain intensity as high [14].

In general, the characteristic symptoms accompanying migraine (e.g., nausea, vomiting, phonophobia, photophobia, cutaneous allodynia) are more frequent among women than men [4, 10–13]. Studies have found that, except for vomiting, among patients with MO, nausea, phonophobia, and photophobia are significantly more frequent in women (*p* < 0.001 for all symptoms), whereas among patients with MA, sex-related differences were found for nausea (65.6% in women and 48.6% in men; *p* = 0.049) and phonophobia (70.2% in women and 51.4% in men; *p* = 0.033) but not for vomiting and photophobia [9].

MA is less frequent than MO in both men and women. A UK study [15] reported a MA 1-year prevalence of 5.8% (2.6% in men and 7.7% in women). MA is more prevalent among women (range, 2.6–10.8% in women and 1.2–3.7% in men) [16]. The frequency of symptoms accompanying aura differs between the sexes: visual aura (1.8% in men and 4.2% in women); sensorimotor aura (0.3% in men and 1.7% in women); and visual and sensorimotor aura combined (0.4% in men and 1.9% in women) [15].

The Global Burden of Disease study (2015) classified migraine as the fourth leading cause of years living with disability (YLD) for women and the eighth cause among men [17], indicating that disability due to migraine is another sex-related factor. Women are 1.34 times more likely (95% CI 1.21–1.48) than men (12.4% for women and 9.3% for men) to report grade IV disability due to migraine in the past 3 months on the Migraine Disability Assessment (MIDAS) questionnaire. Women are also more likely to report inability to carry out household chores (odds ratio [OR] 1.5, 95% CI 1.44–1.56), to take part in social or family activities (OR 1.11, 95% CI 1.06–1.17), and to reduce by at least 50% work or school activity for at least 1 day due to migraine [10]. Studies consistently report greater migraine-related disability in women: 34% of women and 25% of men stated that they lost work or school time more than rarely. Further, over 45% of patients who reported having never or only rarely missed work or school days due to migraine stated that they needed 2 h of bed rest on average [12]. Whereas women reported that they were unable to resume their daily activities for 3 to 6 days after an attack, men stated that they resumed activities on the second day after an attack [10].

The impact of migraine-related disability is especially critical for the toll it takes on the quality of life of migraine sufferers besides the social costs it incurs. A US study published in 1999 [18] reported that women generated about 80% of medical costs directly correlated with migraine treatment and that the indirect costs correlated with lost productivity were 50% higher for women than for men. These data are underestimated since they do not take into account unemployment and underemployment rates due to migraine. The estimated annual mean pro capita cost of migraine is €1222 (95% CI 1055–1389) in Europe, 93% of which are indirect costs. No data on sex-related differences in costs are available [19].

Overall, these sex-related differences in the clinical and epidemiological aspects of migraine translate into differences in migraine treatment between men and women. An Italian survey of patients who purchase headache pain relievers at a pharmacy revealed that men consult a doctor less often than women for treatment (65.7% and 72.4%, respectively; *p* = 0.003; OR 0.71, 95% CI 0.57–0.89), whereas women are more likely to talk with their primary provider (40.5% and 35.9%, respectively; *p* = 0.082; OR 1.211 95% CI 0.976–1.503) or contact a headache center (21.7% and 17.4%; *p* = 0.004, OR 1.31 95% CI 1–07-1.72) [20]. Unlike men, women are more likely to visit the emergency department because of intense migraine pain. These data are consistent with the data for the percentage difference between the sexes in medications use for acute migraine attack or prophylaxis. Compared with men, women use more prescription medications (PR 1.33, 95% CI 1.23–1.43) and are more likely to use triptans (OR 1.41, 95% CI 1.12–1.78) or drug combinations (OR 1.49, 95% CI 1.04–2.14) but less likely not to take acute medications (PR 0.65, 95% CI 0.52–0.80) [10, 11, 13]. In contrast, no significant difference between the sexes was found for the use of nonsteroidal anti-inflammatory drugs or ergot derivatives (55.6% vs. 51.6% and 8.7% vs. 9.3%, respectively) [20]. Women are more likely than men to use preventive treatment (OR 1.37, 95% CI 1.27–1.48). While guidelines for the acute and preventive management of migraine make no distinction between the sexes (except for menstrual migraine treated with hormone therapy), sex differences in the pharmacokinetics of triptans are well documented: peak plasma concentration (Cmax) and area under the curve (AUC) of plasma concentration are higher in women [21]. The clinical relevance of these sex-related differences in triptans pharmacokinetics is controversial since studies have found no correlation between these data and patient response. A study comparing four different triptans found no significant difference in treatment response or recurrence rates at 24 and 48 h between men and women [22].

Data disagree on the likelihood that female sex is a risk factor for transition from episodic to chronic migraine. Some studies have reported that the transition in women is more likely (OR 2.9 95% CI 1.2–6.9) [11], while others have found a nearly similar transition rate within 1 year (5.4% in men and 4.4% in women) [23].

Compared with men with migraine, women migraineurs have more comorbidities (average, 11 and 5 comorbidities in women and men, respectively) and more mental comorbidities (e.g., anxiety and depression), whereas men have more somatic disorders (e.g., obesity). The data for these differences are discrepant, however. Restless legs syndrome is associated with migraine and is more common among women than men in the general population; however, some studies have reported a greater risk for developing the syndrome in men migraineurs [24].

Migraine is a recognized risk factor for the development of cardiovascular disease. Because most studies have involved female subjects, the data for males are scarce. The risk of ischemic stroke is twofold higher for women with MA. The data for defining the risk of hemorrhagic stroke in men are insufficient. The incidence of myocardial infarction seems to be higher among migraineurs than nonmigraineurs (OR 1.33, 95% CI 1.08–1.64), but small male sample size precludes reliable analysis of sex-related differences. MA is associated with a higher risk of venous thrombosis in patients under age 55 years (adjusted hazard ratio [aHR] 3.322, 95% CI 1.509–7.312), specifically among women (aHR 2.81, 95% CI 1.41–5.58 for women and aHR 1.81, 95% CI 0.72–4.55 for men) [11, 25].

Numerous factors underlie the sex-related differences in migraine characteristics: hormonal, genetic, epigenetic, and environmental aspects contribute in different ways to brain structure, function, and plasticity.

## Migraine pathophysiology in female

### Sex hormones

Estrogens and progesterone regulate a host of biological functions through two mechanisms: nongenomic and genomic [26, 27]. Owing to their lipophilic nature and low molecular weight, sex hormones can cross the blood-brain barrier, resulting in similar concentrations in systemic and cerebral circulation [28]. The diverse enzymes involved in sex hormone production are also found in the brain [29].

Three nongenomic mechanisms by which sex hormones influence the central nervous system and its excitability are distinguished: modulation of the interaction between receptors and ligands; alteration of conductance of ion channels through allosteric modulation; and increase in neuron excitability after acute exposure [30].

The genomic mechanisms involved in the central action of sex hormones are explicated through the activation of two estrogen receptors (ERα and ERβ) and two progesterone receptors (PR-A and PR-B). They modulate the production and metabolic pathways of various neurotransmitters and hormones: calcitonin gene–related peptide (CGRP), serotonin, glutamate, noradrenalin, nitric oxide (NO), and endogenous opioids. Progestin receptors often co-localize with estrogen receptors, and their expression may depend on the estrogen receptors; they appear to exert synergistic, antagonistic, or neutral action, depending on the area and the function evaluated [31].

In addition to their central action, sex hormones exert rapid modulation of vascular tone, without altering gene expression, through nongenomic mechanisms [32]. In general, female sex hormones exert a protective action on the cardiovascular system through the inhibition of vascular tone, as demonstrated in different body districts and animal species [33–35]. This action is documented for both estrogens and progesterone [36]. 17β estradiol induces vasodilatation through genomic and nongenomic mechanisms: (1) the production of vasodilating substances such as NO, cyclic guanosine monophosphate, cyclic adenosine monophosphate, and prostacyclins; (2) alteration of the expression of their receptors; and (3) alteration of ion channel activity (calcium and potassium in particular) [33]. Although endothelial factors may be involved in steroid-induced vasodilatation [37], it does not appear to play a preponderant role since vasodilatation occurs also in vessels without endothelium and with NO inhibitor [36, 38, 39]. Summarizing, the vasodilating action of female sex hormones involves multiple cellular mechanisms, depending on type of hormone and type of tissue on which it acts [31].

Female sex hormones modulate the action of many other hormones and vasoactive neuromediators implicated in the onset of migraine. The effects of sex hormones depend on various different factors and can be specific for a certain region or neuron. Estrogens and progesterone can stimulate gene expression in one region but inhibit it in another; they can exert differential actions on the same tissue substrate depending on the hormonal milieu present at a certain moment; different hormone levels can modulate opposite effects; the effect on a system can depend on the hormonal stimulation of other regions connected to it; the action of multiple hormones contemporaneously can differ from the effect of each single hormone; there may be differences depending on whether exposure is acute or chronic; the effect can be agonistic or antagonistic depending on the type of receptor stimulated in the same neurotransmitter [30].

The predominant action of estrogens is to facilitate serotoninergic and glutamatergic tone and to inhibit GABAergic and noradenergic tone, whereas progesterone stimulates GABAergic neurons and modulates the effects of estrogens. Both hormones regulate the pain network and endothelial vasculature [30].

Estrogen administration causes neuronal hyperresponsiveness of the trigeminal nucleus caudalis (TNC), and progesterone causes hyporesponsiveness [40, 41]. During the preovulatory (late follicular) phase (high estrogen, low progesterone levels), glutamatergic and serotoninergic tone is elevated and sympathetic hypofunction is present, whereas the opposite occurs during the mid-luteal phase (low estrogen, high progesterone levels). During the early follicular and late luteal phase (low hormone levels), sympathetic tone is elevated but GABAergic and serotoninergic tone is low. The rise in estrogen levels during the central phase of the cycle increases TNC transmission and sets the stage for periovulatory migraine; however, inhibition by an elevated serotoninergic level could underlie the lower frequency of periovulatory compared with menstrual migraine. During the luteal phase, the increase in progesterone reduces TNC neurotransmission. During the late luteal phase and early follicular phase, the TNC structure changes: the structural changes induced by estrogens in the TNC are not immediately abolished by the reduction and could lead to hyperactivation consequent to the low inhibitory tone due to the decrease in progesterone. This is a plausible explanation for the greater frequency of migraine during menses [30].

### Calcitonin gene–related peptide (CGRP)

Estrogens and progesterone can raise CGRP production and modulate its role in pain transmission [31, 42]. Estrogen therapy was found to increase the release of CGRP at perivascular sensory nerve endings, resulting in the vasodilatation of dural arteries in the rat [43]. In vivo studies in ovariectomized rats showed that treatment with estrogens, progesterone, or both increases CGRP and its mRNA in the dorsal root ganglion [44]. Increased CGRP in the dorsolateral periaqueductal gray (PAG) was found in ovariectomized mice [45].

Blood CGRP concentration is decreased in postmenopausal women [46] and increased in pregnant women [47] and those using contraceptives [48] or hormone replacement therapy [46]. Levels are higher in women than in men [48]. Although elevated CGRP levels seem to be correlated with migraine, the increase during pregnancy is probably due to vasodilatation and vascular adaptation; nonetheless, a distinction should be made between intracerebral and systemic levels [42]. Female sex steroids modulate CGRP levels and mRNA of the receptor and binding sites. Progesterone exerts an opposite action on the effects of estrogens and modulates receptor expression opposite that of estrogens [49].

Experimental models have investigated sex-related difference in the hormone-induced release of CGRP between healthy subjects and migraineurs. In healthy men, cutaneous vessel reactivity was noted to be similar to that of migraineurs, whereas differences in reactivity were correlated with hormonal fluctuations in women, particularly during the perimenstrual phase. In women migraineurs, reactivity was more pronounced probably due to the increased release of CGRP [50]. Studies on the effect of the menstrual cycle on trigeminal-vascular system reactivity were carried out in healthy, postmenopausal women and in migraineurs. Greater reactivity was observed during the menstruation in fertile women [51], not present in the absence of sex hormones during menopause [52]; however, no reactivity of the cutaneous vessels was noted in the migraineurs during menses [53]. In another experimental model investigating the effect of sex hormones, CGRP levels in blood mononuclear cells were measured in women with pure menstrual migraine. In vitro treatment with pharmacological doses of 17β estradiol was observed to increase mRNA levels in the healthy subjects and the migraineurs, whereas CGRP and mRNA levels were decreased after administration of physiological doses only in the migraineurs [52].

### Noradrenalin and α receptors

Estrogens and progesterone can stimulate the release of noradrenalin in the hypothalamus, leading to increased excitability of the ventromedial area [53]. The rate-limiting step for production of noradrenalin is tyrosine hydroxylase, whose synthesis is upregulated by estrogens [30]. Female mice appear to have a lower pain threshold than male mice and to be less responsive to analgesia mediated by α receptor agonists [54]. Estrogens appear to modulate the antinociceptive effect of these receptors: (1) downregulate α2A receptors in the cortex in ovariectomized mice [54]; (2) uncouple α2A receptors from protein G [55]; and (3) attenuate the antinociceptive effect in the spine [56]. Plasma adrenalin levels were noted to be higher in female than in male rats and observed to decrease after ovariectomy [57]. However, estrogens and progesterone reduce sympathetic tone, consistent with observations in migraineurs and the decrease in plasma noradrenalin during menses in migraineurs compared with controls [58].

In addition to the effects on neurons, sex hormones modulate vasoconstriction mediated by α2 receptor agonists, inducing a dichotomic effect depending on the vascular district involved [31]. Studies have shown that infusion of estrogens after ovariectomy reduces α2 receptor-mediated vasoconstriction [59]. Increased α2 receptor function may be partly responsible for elevated blood pressure during postmenopause [60]. Other data showed a vasoconstrictive effect of estrogen replacement therapy in ovariectomized mice mediated by α2 receptors, which could be masked by an endothelial influence, particularly by NO [61].

Vascular sensitivity to noradrenalin is regulated in opposite directions by estrogens and progesterone. Female sex hormones can act on noradrenergic receptors, such as releasing noradrenalin in diverse brain areas. The subsequent increase in cortical excitability, inhibition of antinociceptive tone, and modulation of vascular tone are predisposing factors for migraine in women [31].

### Serotonin

Female sex hormones can modulate the synthesis at both central and vascular levels. Neurophysiological studies on primates have demonstrated a link between estrogen and serotonin mediated by the ERβ receptor [31].

In their study, Nappi et al. used meta-chlorophenylpiperazine (m-CCP), a 5-HT receptor agonist, to evaluate the involvement of 5-HT receptor in the occurrence of status migrainosus triggered by discontinuation of combined oral contraceptives (COCs). They demonstrated that status migrainosus is associated with impaired neuroendocrine response to 5-HT challenge compared with healthy controls and transdermal estrogen delivered during the COCs interval improved migraine symptoms and restored 5-HT response [62]. Also, the firing rate of serotoninergic neurons in the nucleus of the dorsal raphe is higher in male than female rats [63]. Estrogens stimulate tryptophan hydrolase, the rate-limiting enzyme in the synthesis of serotonin; this effect does not seem to be modified by the addition of progesterone [64]. The serotonin reuptake transporter (SERT) is also modulated by estrogens, the effect of which depends on the duration of hormone therapy. The degradation enzymes MAO-A and MAO-B are inhibited by estrogens and progesterone [30]. In vitro studies on the vasoconstrictive action of serotonin have shown a reduction after exposure to estrogens in diverse vascular districts through a direct effect on muscle cells but not on the endothelium. Progesterone appears to mediate similar effects alone and in combination with estrogens [31].

### Ion balance

Magnesium and calcium levels are influenced by sex steroids and vary across the menstrual cycle through genomic and nongenomic mechanisms. Thus, an increased neuron excitability is caused by an inverse correlation between estrogen and progesterone and cytoplasmic Mg levels. Calcium levels increase with increasing estrogen levels. Calcium and potassium ion channels are coupled with estrogen receptors through protein G, which are activated by sex hormones. It remains to be established whether these modulations are relevant in migraine pathophysiology [31].

### Nitric oxide (NO)

Estrogens can directly influence vascular tone by increasing the secretion of NO and its synthesis by stimulating NO synthetasis in the endothelium [65]. NO and platelet L-arginine pathways are active in women with menstrual migraine, particularly during the luteal phase, compared with women not affected by menstrual migraine and healthy women [66]. Studies in rats showed that estrogens reduce arterial tone via a NO-dependent mechanism. The interaction between sex hormones and nuclear or cytosolic receptors triggers genomic effects that can lead to endothelial cell growth and inhibit the growth of smooth muscle cells. Nongenomic effects can also occur, such as stimulation of NO or prostacyclin synthesis [31]. In postmenopausal women, vascular function can be improved with estrogen valerate, which increases NO levels, whereas combination with medroxyprogesterone acetate reduces the amount of increase [67].

### GABA

Activation of GABAergic receptors induces neuroinhibition, which is influenced by female hormones. The GABA_A_ receptor opening time is lengthened by stimulation by allopregnanolone, leading to hyperpolarization [68]. Differently, estrogens uncouple the receptor and the G protein-coupled inward rectifier K+ (GIRK) channels, which reduces receptor function and increases depolarization [69]. Besides their central action, sex steroids act on GABAergic receptors in vascular smooth muscle. GABA is one of the weakest vasodilating substances. However, there is other evidence for estrogen action that estrogens promote GABA synthesis and its release and synapse formation [31].

### Glutamate

Estrogens and progesterone differ in the effects on glutamate receptors depending on the brain area where they act. Acute estrogen exposure increases the firing rate of Purkinje cells after the application of glutamate [30]. Estrogens promote glutamatergic transmission (N-methyl-d-aspartate [NMDA] and kainate) in the hippocampus [70], but the same effects are not observed in other brain areas [70]. In contrast, progesterone inhibits NMDA receptors in the frontal cortex [70]. Glutamate receptors appear to be involved in the peripheral release of CGRP [31].

### Endogenous opioids

Sex-related differences in morphine-induced analgesia have been documented in numerous animal species, including humans [71]. Antinociceptive activation is greater in men than in women. During pregnancy, enkephalin and dinorphine levels are increased, as are levels of estrogens and progesterone [72]. Estrogens and progesterone increase the expression of mRNA of pro-opiomelanocortin. Their effects differ depending on the type of receptor and brain area [31].

## Brain structure and function

Imaging studies have shown considerable sex-related differences in the gray and white substance, in numerous functional networks, and in brain biochemistry. The number of anomalous functioning connections is greater, and the degree of resilience against function loss of brain networks is lower in women [73]. Differences are also related to the menstrual cycle: the gray matter is thicker during the ovulatory phase. In women using hormonal contraceptives, the volume of gray matter at the frontal cortex, the pre- and postcentral gyro, the parahippocampus, and the temporal region is greater than in women with a normal hormonal cycle. Hormonal fluctuations influence the perception of pain, which may increase susceptibility to migraine attacks at certain points in the menstrual cycle [74].

Estrogens modulate neuron activity by stimulating the ER distributed throughout numerous brain areas, many of which are implicated in the onset of migraine (e.g., the pulvinar, the accumbens nucleus, and the amygdala). Changes in the volume or ratio between white and gray matter depending on cycle phase were observed in the hippocampus, the basal ganglia, and the amygdala [74].

Greater thickness of the posterior insula and the precuneus was noted in women migraineurs compared with men and healthy controls. Stimulation of heat-induced pain elicits sex-related differences in response patterns. Based on this observation, a sex phenotype for migraine has been hypothesized in which sensory and emotional circuitry differs between men and women [75].

## Conclusion

Several factors may modulate female predisposition to migraine throughout genetic and epigenetic mechanisms: hormones, brain structure, genetic polymorphism or mutation, life events, stress, and neuronal activity. All these are cross-connected, and they influence each other. This specific sex phenotype for women should be considered during clinical management and experimental studies.
